# Potential applications of nanotechnology for the diagnosis and treatment of pancreatic cancer

**DOI:** 10.3389/fphys.2014.00002

**Published:** 2014-01-24

**Authors:** Joshua McCarroll, Joann Teo, Cyrille Boyer, David Goldstein, Maria Kavallaris, Phoebe A. Phillips

**Affiliations:** ^1^Tumor Biology and Targeting Program, Lowy Cancer Research Centre, Children's Cancer Institute Australia, University of New South WalesSydney, NSW, Australia; ^2^Australian Centre for NanoMedicine, University of New South WalesSydney, NSW, Australia; ^3^Panceatic Cancer Translational Research Group, Lowy Cancer Research Centre, Prince of Wales Clinical School, University of New South WalesSydney, NSW, Australia

**Keywords:** pancreatic cancer, nanotechnology, nano-diagnostics, molecular imaging, therapeutic drug delivery, tumor stroma

## Abstract

Despite improvements in our understanding of pancreatic cancer and the emerging concept of personalized medicine for the treatment of this disease, it is still the fourth most common cause of cancer death in the western world. It is established that pancreatic cancer is a highly heterogeneous disease with a complex tumor microenvironment. Indeed the extensive stroma surrounding the cancer cells has been shown to be important in promoting tumor growth and metastases, as well as sequestering chemotherapeutic agents consequently decreasing delivery to the tumor cells. Nanotechnology has come to the forefront in the areas of medical diagnostics, imaging, and therapeutic drug delivery. This review will focus on the potential applications of nanotechnology for diagnosis, imaging, and delivery of therapeutic agents for the treatment of pancreatic cancer.

## Introduction

Pancreatic cancer is one of the most lethal cancers worldwide with a 5-year relative survival rate of <6% (Jemal et al., 2011; Siegel et al., 2013). Despite aggressive combinations of therapies ranging from surgery, chemotherapy, and radiotherapy, patients diagnosed with this malignancy have extremely high mortality rates and poor prognosis (Vincent et al., 2011). These dismal outcomes can in part be attributed to a lack of early diagnosis and inability to detect pre-cancerous pancreatic intraductal neoplastic (PanIN) lesions, which often give rise to invasive pancreatic tumors (Misek et al., 2007). Currently no clinically reliable serum biomarkers for early detection and diagnosis at an early stage of pancreatic cancer are available (Misek et al., 2007). A majority of pancreatic cancers are diagnosed and staged by computed tomography (CT) imaging or magnetic resonance imaging (MRI) with a tumor detection limit of 5–8 mm when the earliest precursor lesions are in the microscopic range (Holzapfel et al., 2011; Canto et al., 2012). Consequently, >80% of patients have locally advanced or distant metastases upon diagnosis, with chemotherapy or palliative chemotherapy being the current standard for systemic treatment (Vincent et al., 2011). Notably, only 20% of patients at the time of diagnosis are suitable for potentially curative pancreatectomy and despite combination with adjuvant chemotherapy, a majority of patients still develop local recurrences and systemic metastases that results in mortality (Hidalgo, 2010). Another contributing factor to the poor outcome of pancreatic cancer is that the current standard first-line therapy of the chemotherapeutic agent gemcitabine shows only limited efficacy by extending the overall survival of patients by only 6–12 weeks (Hidalgo, 2010). This is partially due to poor understanding of the biology of the individual patients' tumor, chemotherapy resistance and the tumor microenvironment.

Nanotechnology is defined as the manipulation of organic or inorganic materials to form structures on the scale of nanometers. Recently, advances in nanotechnology have provided great opportunities for strategies in advancing cancer diagnostics, imaging, and therapeutic drug delivery (Schroeder et al., 2011; Melancon et al., 2012; Prabhu and Patravale, 2012). Nanoparticles have the potential to not only increase the efficacy per dose of a therapeutic or imaging contrast formulation by increasing its bioavailability, but can also be modified for targeted selectivity toward tumor cells to increase image resolution and/or reduce off-target toxicities associated with current chemotherapy (Ferrari, 2005). Furthermore, they show promise in treating metastatic cancers like pancreatic cancer [see detailed review (Schroeder et al., 2011)]. Nevertheless, in order to design effective nanoparticles to deliver either imaging contrast agents or therapeutics (by passive or active targeting) requires a thorough understanding of the physiological barriers specifically associated with the disease and apply nano-engineered components for effective nanoparticle extravasation, accumulation and penetration into the tumor (Jain and Stylianopoulos, 2010). This review highlights promising translational prospects and the challenges of integrating nano-engineering sciences with pancreatic cancer biology to develop nanomaterials that could enhance diagnostics, imaging, and therapeutics against this devastating malignancy (Table 1).

**Table 1:** **Potential advantages of nanotechnology for the diagnosis and treatment of pancreatic cancer**.

**Advantages of nanotechnology in diagnostics and imaging for pancreatic cancer**	**Advantages of nanotechnology for therapy in pancreatic cancer**
Increased sensitivity and specificity compared to conventional assays using only small amounts of patient sample.	Increased drug delivery to tumor cells.
Detection of early cancer biomarkers in blood samples (RNA/DNA, exosomes, proteins).	Increased tumor specificity via the use of tumor cell targeting moieties.
Monitor patient treatment response via biomarker detection and/or imaging.	Potential to decrease off-target systemic drug toxicity.
Potential to non-invasively differentiate between tumor and stromal elements in pancreatic cancer.	Potential to deliver therapeutics to target and silence non-druggable genes using RNAi inhibitors.
Increased sensitivity to detect small local and distant metastases.	Provide increased solubility, stability and circulation half-life for current chemotherapeutic drugs.

## The pancreatic tumor microenvironment and its role in promoting chemotherapy resistance

Desmoplasia and hypovascularity are the pathological hallmarks of pancreatic tumors. The desmoplastic microenvironment (also known as the stroma) can make up >90% of the tumor mass (Li et al., 2010; Neesse et al., 2011). The stroma contains a number of different cell types including, pancreatic stellate cells (PSCs), endothelial cells, immune cells and dense extracellular matrix (ECM) (Li et al., 2010; Neesse et al., 2011). Indeed, it is well established that PSCs are the principle cell type responsible for the production of stromal fibrosis (Apte et al., 2004; Bachem et al., 2005). Importantly, the extensive fibrotic characteristic of pancreatic cancer results in reduced intratumoral vascular density which give rise to compromised dysfunctional vessels that cause a decrease in blood flow; inadequate venous and lymphatic drainage also further increases the interstitial fluid pressure within pancreatic tumors (Koong et al., 2000; Erkan et al., 2007; Komar et al., 2009; Olive et al., 2009). As a consequence the stroma has been shown to play a major role in poor chemotherapy drug delivery, penetration, and rapid metabolic inactivation of therapeutic agents which contribute to an unusually poor response to treatment (Olive et al., 2009; Provenzano et al., 2012). Furthermore, the bi-directional interaction that occurs between PSCs and the tumor cells further potentiates tumor progression, chemoresistance, invasion, and metastases (Apte et al., 2004; Bachem et al., 2005; Vonlaufen et al., 2008; Olive et al., 2009; Xu et al., 2010; Phillips, 2012). However, the stroma could also be the achilles' heel for targeted diagnostic imaging and therapeutic drug therapy to ablate the microenvironment that supports tumor growth and metastases (Phillips, 2012; Heinemann et al., 2013). In addition, given the tumor mass comprises mainly of the fibrotic stroma, the ECM components or PSCs residing within the stroma could be novel targets for early diagnosis, imaging, and targeted therapy. Examples of how nanotechnology may be applied to advance pancreatic cancer diagnosis and treatment are described below.

## Nano-diagnostics for the early detection of pancreatic cancer

The vast majority of long-term survivors of pancreatic cancer (> than 5 years) have resectable disease upon diagnosis and are suitable for curative surgery, suggesting early detection and intervention may increase the overall survival of patients (Slavin et al., 1999). However, currently there are no reliable serum biomarkers with the sensitivity and specificity to accurately detect early pre-cancerous lesions (Goggins, 2005). This is largely due to the lack of pancreatic cancer biomarkers able to distinguish between a benign diseased pancreas, such is the case for chronic pancreatitis and cancer, as both are hard to distinguish under current imaging modalities (Erkan et al., 2012a,b). Moreover, the heterogeneous nature of pancreatic cancer and the complex stromal microenvironment also present a challenge for potential biomarkers. Hence, early diagnosis of pancreatic tumors may require the simultaneous identification of a panel of biomarkers to have greater accuracy compared to single biomarkers.

Nano-diagnostic platforms have the potential to revolutionize the cancer diagnostics field by developing faster, more accurate, cost-effective, and reliable biomarker detection systems with lower detection limits (Cao, 2008; Chikkaveeraiah et al., 2012; Malhotra et al., 2012). Various electrochemical immunosensors have also integrated nanomaterials such as magnetic particles, gold nanoparticles, quantum dots, and carbon nanotubes to increase their sensitivity for electrochemical detection of tumor biomarkers(Cao, 2008; Chikkaveeraiah et al., 2012; Malhotra et al., 2012). For instance, microfluidic biochips integrated with highly luminescent quantum dots have shown promise as a versatile multicolor and multiplexed bioassay(Hu et al., 2010). This was evidenced by their ability to rapidly detect two cancer biomarkers, carcinoma embryonic antigen, and α-fetoprotein, with high selectivity and femtomolar sensitivity in human serum (Hu et al., 2010). Recently, Chang et al. (2011) managed to design passivated (technique used to prevent or retard non-specific background signal) nanowire biosensors to allow rapid and specific detection of the ovarian cancer biomarkers, cancer antigen-125 and insulin-like growth factor-II directly from human whole blood collected simply from a finger prick, with much lower limits of detection compared to the clinically relevant levels for diagnosis (Chang et al., 2011). Others like, the NanoMonitor incorporated nanoporous alumina membranes onto micro-fabricated silicon platforms allowing rapid label-free analysis of glycans from pancreatic cancer cell lysates with high sensitivity and selectivity (Nagaraj et al., 2010). Biosensors like these could be useful for pancreatic cancer diagnosis in the clinic by identifying other transmembrane glycoproteins such as mucins (MUC) which are expressed on the surface of pancreatic cancer cells and are important regulators of tumor growth and metastases (Kaur et al., 2013). Indeed, studies have demonstrated several members of the MUC protein family to be overexpressed and/or aberrantly glycosylated during the progression of early PanIN lesions to pancreatic tumors in a mouse transgenic model (Kras^G12D^;Pdx1-Cre), which mimics the human setting for the initiation and progression of pancreatic cancer (Rachagani et al., 2012; Remmers et al., 2013). Nano-diagnostics could also be explored in the case of pancreatic cancer-induced paraneoplastic diabetes to aid early diagnosis (Sah et al., 2013). It has been suggested that up to 85% of pancreatic cancer patients have diabetes or hyperglycemia which can manifest as early as 2–3 years before the development of pancreatic cancer (Sah et al., 2013). Hence, nano-diagnostics which employ the use of a combination of early cancer biomarkers along with indicators of β cell dysfunction (resulting in worsening type 2 diabetes and increased adrenomedullin levels) could have potential diagnostic utility, to be explored in a large patient cohort (Hart et al., 2011; Aggarwal et al., 2012; Sah et al., 2013). In summary, nano-diagnostics are powerful tools for cancer detection, prevention, and diagnosis. Nano-diagnostic platforms may provide additional sensitivity with relatively small sample volumes for biomarker detection. Furthermore, the increase in sensitivity, specificity, and multiplex properties for the detection of cancer biomarkers using nano-diagnostics could also be used as a monitoring platform to detect early recurrences or to follow the response of patients to treatment that would otherwise be undetectable with conventional assays.

## Nanomaterials for advancing pancreatic cancer imaging

Currently, the most accurate initial screening modalities for the detection of pancreatic cancer are endoscopic ultrasonography (EUS) and/or MRI cholangiopancretography (MRCP) (Hekimoglu et al., 2008; Hyodo et al., 2012). The limitation of current imaging-based screens is that upon identification of potentially benign cysts/lesions, subsequent invasive evaluation by collecting tissue biopsies (for example through fine needle aspiration) is still required to confirm diagnosis (Brand, 2001). Early diagnostics to identify carcinomas *in situ* like PanIN lesions are often challenging due to the detection limit of current radiological methods and how chronic pancreatitis masses show similar pathological aspects (fibrotic stroma) to pancreatic cancer (Erkan et al., 2012a,b). Only cystic tumors are detectable at a pre-invasive stage with conventional radiological methods due to their large size and high contrast compared to the normal pancreas (Erkan et al., 2012a,b). Furthermore, smaller (<1 cm) metastatic tumor deposits in the liver and/or peritoneal cavity may be overlooked by MRI or CT.

To improve the detection limit of MRI or CT-imaging, an extraneous imaging agent can be administered to the patient to enhance the imaging signals leading to the commercial development of a large range of small organic contrast agents. For example, more than one third of MRI scans are performed in conjunction with a contrast imaging agent. The first generation of organic contrast agents had some limitations (poor specificity and rapid renal clearance) (Roberts, 1997; Kim et al., 2011). To overcome these limitations, contrast agents have been designed in a nanoparticle formulation (Hahn et al., 2011). The incorporation of contrast agents into nanoparticles can offer significant advantages, such as easy functionalization by targeting moieties, higher sensitivity compared to small organic contrast agents and improved biodistribution. For instance, inorganic nanomaterials [including gold nanoparticles, and superparamagnetic iron oxide nanoparticles (SPION)] have been investigated as potential contrast agents for cancer imaging (Godin et al., 2011; Huang et al., 2011). Gold and iron oxide nanoparticles (IONPs) in particular offer several advantages compared to conventional small organic MRI and CT contrast agents (Godin et al., 2011; Huang et al., 2011). For example, IONPs have a lower toxicity profile when compared to gadolinium based contrast agents used in current MRI (Boyer et al., 2010). These nanoparticles have the potential to enhance the contrast between the delayed uptake (hypoperfusion) of the hypovascular tumors when compared to the normal parenchyma during the arterial and venous phase with conventional radiology approaches (Erkan et al., 2012a,b). Indeed, various nanoparticle systems have been generated with enhanced longitudinal relaxation time (*T*_1_) and transverse relaxation time (*T*_2_) for high spatial resolution and simultaneous extraction of physiological, molecular, and anatomical information using MRI. Gadolinium-based contrast nanoparticles have been investigated to enhance *T*_1_-weighted imaging; while various iron oxides, alloyed and bimetallic ferrite nanoparticles have been shown to be promising *T*_2_ contrast agents (Huang et al., 2011). In a study by Kumagai et al. (2010) the authors designed and synthesized a high-density pegylated-coated iron oxide-gold core shell nanoparticle for MRI imaging. The nanoparticle was approximately 25 nm in diameter. Systemic administration of the nanoparticle to mice bearing a subcutaneous colon tumor or an orthotopic pancreatic tumor resulted in its high accumulation into tumor tissue and low non-specific accumulation in the liver and spleen. In another example, extremely small sized (<4 nm) IONPs have been used as *T*_1_ contrast agents with further improvements in reducing non-specific toxicity and enhanced properties for overcoming the commonly observed “blooming effect” (a phenomenon observed which exaggerates the size of a labeled area as well as blurs the image) of *T*_2_ contrast agents (Kim et al., 2011). In addition, multifunctional nanoparticles or hybrid systems have also shown great promise. These nanomaterials possess greater signal amplification further improving the diagnostics and imaging sensitivity, while also having the capacity to be used as a therapeutic. For instance, Kirui et al. (2013) reported the use of a gold-iron oxide hybrid nanoparticle to image and treat colorectal tumors in mice. The iron oxide enabled for MR imaging of the tumor, while the gold allowed for photothermal therapy. To improve tumor imaging and targeted therapy the authors conjugated an antibody to the surface of the nanoparticle which recognized the A33 antigen which is expressed on the surface of colorectal tumor cells (Kirui et al., 2013). Importantly, administration of the hybrid nanoparticle to mice with xenografted colorectal tumors showed highly effective tumor imaging and increased tumor cell death when laser irradiation was applied (Kirui et al., 2013). In another example, Kaida et al. (2010) demonstrated the use of a supramolecular nanocarrier system which incorporated a clinically approved gadolinium-based MRI imaging contrast agent and a platinum anti-cancer drug (oxaliplatin). The authors showed successful imaging and combined anti-tumor therapy using the supramolecular nanoparticle in an orthotopic pancreatic cancer mouse model without any significant off-target toxicity (Kaida et al., 2010). Moreover, the amount of gadolinium delivered by the nanoparticle to the tumor was seven times higher when compared to free-gadolinium alone (Kaida et al., 2010). However, it is to be noted, that most nano-formulated contrast agents depend on passive targeting to the tumor site via the enhanced permeability and retention (EPR) effect (Cheng et al., 2013). The EPR effect was first described in 1986 by Matsumura and Maeda (1986) and is a result of rapid and uncontrolled angiogenesis during solid tumor growth which gives rise to leaky and damaged vasculatures within the tumor microenvironment compared to normal tissue (Maeda et al., 2000). This phenomenon allows for vascular extravasation of nanoparticles from a wide size range (100–700 nm) to solid tumors. However, it is to be noted that nanoparticles which enter the solid tumor do not necessarily bind specifically to tumor cells, and have the potential to be taken up by other cell types in the tumor microenvironment (Olive et al., 2009; Erkan et al., 2012a,b).

Nevertheless, despite potential pitfalls with passive tumor targeting, nanotechnology has the ability to advance molecular-targeted imaging in pancreatic cancer owing to the ease of functionalizing nanoparticle surfaces with targeting moieties (antibodies, aptamers, and small molecules) to provide enhanced binding affinity and specificity toward the tumor. This was recently illustrated in a study by Yang et al. (2009) which demonstrated the use of multifunctional nanoparticles to target a cell surface receptor urokinase plasminogen activator receptor (uPAR) which is highly expressed in pancreatic cancer cells and tumor stromal cells. Importantly, the nanoparticles enhanced visualization of pancreatic tumors with a high level of sensitivity in an orthotopic pancreatic cancer mouse model using either non-invasive near-infrared optical imaging or MRI (Yang et al., 2009). In another study, a peptide phage display library was used to screen for small peptides which selectively bound to the surface of pancreatic tumor cells in a genetically-engineered mouse model of pancreatic ductal adenocarcinoma (Kelly et al., 2008). Using this technology the authors identified a small peptide which was able to distinguish the tumor cells from surrounding normal pancreatic ductal cells. Furthermore, proteomic analysis revealed that the peptide bound to plectin-1 (intermediate filament of the cell cytoskeleton). The peptide was then conjugated to the surface of magneto-fluorescent nanoparticles and in conjunction with intravital microscopy and MRI imaging the nanoparticles were able to selectivity detect small pancreatic tumors and pre-cursor lesions with high sensitivity in the mouse model (Kelly et al., 2008). This study highlights the potential of targeted nanoparticles to allow for the visualization of molecular markers that identify specific stages of pancreatic tumor development. More recently, attempts have been made to identify potential markers for the active targeting of pancreatic stroma. Directing contrast agents to the stromal components of pancreatic cancer may amplify contrast signals at both the tumor and precursor lesion sites. For example, Erkan et al. (2007) showed that periostin a secretory protein that accumulates in fibrotic areas is exclusively produced by activated PSCs and is highly expressed in pancreatic cancer compared to normal pancreatic tissue. Thus, the exclusive expression of periostin in the pancreatic tumor microenvironment may be a potential novel target for molecular imaging of pancreatic cancer (Erkan et al., 2007). This concept was recently illustrated by Eck et al. (2008) which took advantage of the strong light scattering signal from gold nanoparticles. To target tumor stroma the nanoparticles were conjugated with antibodies directed against fibroblast activation protein-α which is produced specifically by activated fibroblasts in tumor stroma (Eck et al., 2008). Taken together, these studies highlight the potential for the combined use of molecular markers which target pancreatic cancer cells and/or the surrounding tumor stroma and nanotechnology to improve the specificity and sensitivity of current pancreatic cancer imaging modalities.

## Nanomedicines as a novel class of therapeutics for pancreatic cancer

The design and synthesis of nanoparticles which can encapsulate and deliver a diverse range of therapeutic compounds—ranging from chemotherapy agents to DNA/RNA has received significant attention in cancer research. Nanoparticles in the form of liposomes and/or polymer-derived nanomaterials have been widely used in a number of pre-clinical cancer models. Importantly, these nanoparticles have shown great potential as highly efficient delivery vehicles for chemotherapy drugs or RNA interference (RNAi) inhibitors and are currently being evaluated in human clinical trial (Blanco et al., 2011; Namiki et al., 2011; Schroeder et al., 2011; Singh et al., 2012). A select number of examples for the use of nanoparticles to deliver chemotherapy agents or RNAi inhibitors are described in the following sections.

### Nanoparticles as delivery vehicles for chemotherapy drugs

Many chemotherapeutic agents are associated with debilitating off-target toxicity, poor tumor bioavailability, and unfavorable pharmacokinetics. One strategy to overcome these challenges is the use of nanotechnology as efficient carriers for chemotherapeutic drugs. The rational design of nanoparticles for chemotherapeutic drug delivery has enabled the improved solubilization of the drug, as well as increased its stability and half-life in circulation (Blanco et al., 2011; Pearce et al., 2012). In addition, nanoparticles encapsulated with chemotherapy agents are able to avoid multi-drug resistant efflux pumps expressed on the surface of most tumor cells (Blanco et al., 2011; Pearce et al., 2012). The first nanoparticle-drug approved by the FDA (1995) was “Doxil.” Doxil is a liposome with an approximate size of 100 nm in diameter which encapsulates the chemotherapy drug doxorubicin (Barenholz). To improve tumor bioavailability the liposome was modified to contain a small amount of polyethylene glycol (PEG)-lipid to reduce the clearance of the nanoparticles from the blood, and increase the plasma half-life of doxorubicin. Indeed, encapsulation of doxorubicin within the liposome was shown to significantly alter its pharmacokinetic and pharmacodynamic properties leading to increased tumor uptake and anti-cancer activity, along with a reduction in systemic off-target toxicity (Barenholz, 2012). Today Doxil is used to treat a number of different solid tumors, including platinum-resistant ovarian cancer (Leamon et al., 2013).

Over the recent years, there have been attempts to develop nanoparticle formulations of the chemotherapeutic agent gemcitabine. Indeed, gemcitabine has long been the first-line treatment for patients with unresectable locally advanced or metastatic pancreatic cancer (Vincent et al., 2011). However, despite its use as a first-line treatment, patient survival has only been extended by 6–12 weeks (Hidalgo, 2010). Hence, in an attempt to improve the delivery of gemcitabine to pancreatic tumors as well as overcome some of the acquired gemcitabine-resistant mechanisms in pancreatic cancer cells nanoparticles have been used as a delivery vehicle. Recently, Wonganan et al. (2013), Zhu et al. (2013) demonstrated the potential of a nanoparticle encapsulated with the pro-drug of gemcitabine [4-(N) stearoyl gemcitabine] (GemC18) to overcome resistance associated with ribonecleotide reductase subunit M1 overexpression in pancreatic cancer cells. Interestingly, the authors showed that encapsulation of the gemcitabine pro-drug into the nanoparticle allowed for a different mode of entry into the cell which allowed the gemcitabine to be hydrolyzed more efficiently to its active form compared to the pro-drug alone (Wonganan et al., 2013; Zhu et al., 2013). In another example, Lee et al. (2013) engineered IONPs to express a uPAR-targeted moiety on their surface. Both pancreatic tumor cells and the blood vessels within the tumor stroma have high amounts of the uPAR receptor (Harvey et al., 2003). The magnetic IONPs also had gemcitabine attached via a lysosomally cleavable tetrapeptide linker (Lee et al., 2013). Importantly, systemic delivery of the nanoparticle-gemcitabine complex significantly inhibited the growth of orthotopically xenografted pancreatic tumors in mice and allowed for the detection of residual tumors following treatment using MRI (Lee et al., 2013). More recently, there has also been interest in developing nanotechnology to improve the delivery and efficacy of other chemotherapeutic agents which can be delivered in combination with gemcitabine or used as second-line treatment for pancreatic cancer. For example, Cabral et al. (2013) synthesized micelle nanoparticles which were able to self-assemble with the chemotherapeutic drug oxaliplatin. These nanoparticles were designed to gradually release their contents over time when only exposed to the tumor microenvironment. Importantly, the authors demonstrated that repeated systemic administration of the drug-loaded nanoparticles was able to significantly reduce tumor growth as well as the incidence of metastases in a clinically relevant transgenic mouse model of pancreatic cancer. Therefore, this therapy may be beneficial for treatment of patients with early-stage pancreatic cancer so as to prevent or retard the development of metastases.

Another nanoparticle-bound chemotherapy agent which has generated significant interest is albumin-bound paclitaxel known as Nab-paclitaxel or Abraxane® (Abraxis Bioscience). A recent phase I/II trial for pancreatic cancer demonstrated the maximum-tolerated dose for Nab-paclitaxel in combination with gemcitabine (Von Hoff et al., 2011). The authors also reported an improved overall survival in patients treated with nab-paclitaxel plus gemcitabine (12.2 median months of overall survival) compared to gemcitabine alone. Moreover, Nab-paclitaxel alone and in combination with gemcitabine was shown to deplete pancreatic stroma in pancreatic cancer xenograft mouse models (Von Hoff et al., 2011). Importantly, the depletion of stroma led to a 2.8 fold increase in the intratumoral concentration of gemcitabine (Von Hoff et al., 2011). A recent phase III MPACT (Metastatic Pancreatic Adenocarcinoma Clinical Trial) trial also showed that the addition of Nab-paclitaxel with gemcitabine was not only able to significantly improve the median survival of metastatic pancreatic cancer patients (8.5 months) when compared to gemcitabine treated only arm (6.7 months), but also significantly reduced toxicities (neuropathy and neutropenia) commonly associated with the cremaphor formulation used to dissolve paclitaxel thereby, allowing for a higher paclitaxel dose to be delivered (Ma and Hidalgo, 2013; Von Hoff et al., 2013). The authors also reported an increase in peripheral neuropathy and myelosuppression. However, these side-effects appeared to be reversible (Von Hoff et al., 2013).

Finally, nanotechnology had also been used to improve drug kinetics and tumor bioavailability, of a therapeutic agent by directly targeting and ablating the tumor stroma. Indeed, Provenzano et al. (2012) showed that the desmoplastic reaction surrounding pancreatic tumor cells generated very high amounts of interstitial fluid pressure along with the induction of vascular collapse (Provenzano et al., 2012). Systemic administration of PEGylated human recombinant PH20 hyaluronidase (PEGPH20) [an enzymatic agent that targets a critical component of the desmoplastic stroma in pancreatic cancer known as hyaluronic acid (HA)] (Provenzano et al., 2012) in a murine pancreatic cancer model, produced a marked decrease in tumor stroma which correlated to a rapid and signi?cant decrease in interstitial tumor pressure and increased tumor blood vessel lumen diameter. Furthermore, when delivered in combination with gemcitabine there was a strong anti-tumor effect compared to gemcitabine alone (Provenzano et al., 2012). Collectively, these studies highlight the advances in nanotechnology as highly effective carriers for the passive and active delivery of chemotherapy drugs to tumor cells in pre-clinical and clinical settings. In the future, nanotechnology which can therapeutically target the tumor stroma to enable stroma depletion and tumor vascular normalization as well as deliver therapeutics directly to the tumor cells may become a highly effective novel treatment strategy for pancreatic cancer.

### Nanoparticles as delivery vehicles for RNA interference inhibitors

**Figure 1:**
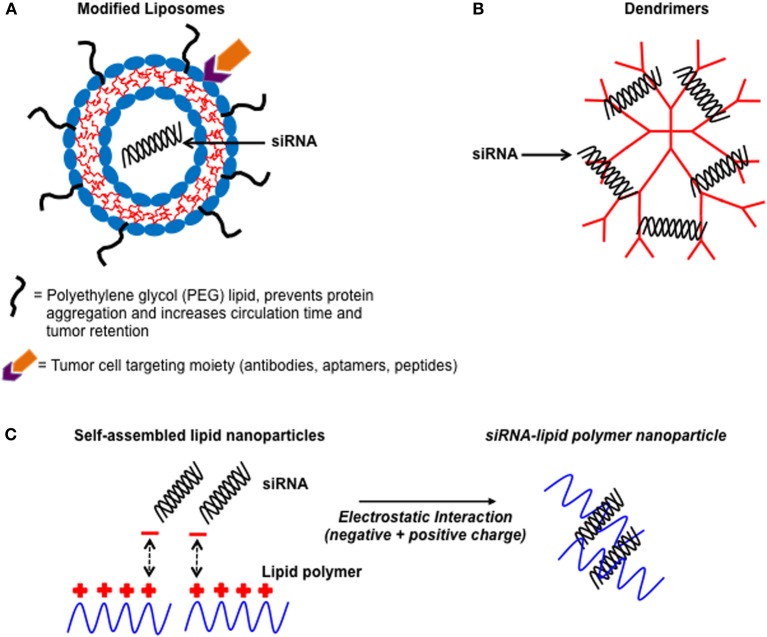
**Illustration showing some common non-viral nanoparticles used as delivery vehicles for siRNA.** A schematic diagram of non-viral nanoparticle-siRNA delivery vehicles. **(A)** Liposomes which have been modified with PEG chains and contain active targeting moieties on their surface to improve tumor bioavailability. **(B)** Dendrimers which are highly branched well-defined structures that complex with siRNA via electrostatic interactions. **(C)** Cationic (positively charged) lipid polymers interacting with negatively charged siRNA to form self-assembled nanoparticles.

RNA interference (RNAi) based therapeutics are emerging as an innovative and promising alternative over conventional systemic treatments in terms of specificity, toxicity, and overcoming multiple drug resistance. RNAi is an endogenous gene-silencing mechanism that can cause the degradation of any mRNA, once the RNA target sequence is known (Rana, 2007). In particular, short-interfering RNAs (siRNA) have gained attention due to their ability to potently silence target gene expression both *in vitro* and *in vivo* (Rana, 2007). siRNAs are processed double stranded RNAs approximately 21 nucleotides in length, and are involved in post-transcriptional gene silencing toward targeted mRNAs. The RNA-induced silencing complex (RISC) located within the cytoplasm of the cell act as a guide for the cleavage of mRNAs bearing a complementary sequence to the siRNA (Rana, 2007). Once activated RISC can be recycled multiple times to cleave additional mRNA targets (Rana, 2007). Importantly, the introduction of chemically synthesized siRNAs into the cell can activate this naturally-occurring mechanism, which can be harnessed as a powerful gene therapy to suppress specific genes associated with human disease, including cancer. However, delivery of siRNA to a target cell remains a challenge. The major limitation of *in vivo* siRNA delivery is its instability and vulnerability to degradation in serum and inability to readily enter a cell due to its high anionic (negative) charge (Baigude and Rana, 2009). Nanoparticles in the form of liposomes, lipid polymers, and dendrimers have been developed over the last decade to act as highly effective siRNA delivery vehicles (Figure 1) (Zimmermann et al., 2006; Baigude et al., 2007; Davis et al., 2010; Su et al., 2011). Indeed, nanoparticles can be designed to self-assemble with siRNA and protect it from serum degradation and elimination from the body (Schroeder et al., 2010). Moreover, nanoparticles can be tailored to possess multifunctional components to allow for targeted siRNA delivery and efficient entry to a specific cell type (Schroeder et al., 2010). The therapeutic potential of nanoparticle-siRNA complexes to treat human disease was first reported by Zimmermann et al. (2006). In this study, the authors used modified nanoparticles (liposomes) known as Stable Nucleic Acid Lipid Particles (SNALP)s to deliver siRNA targeting apolipoprotein B (apo B) (a protein involved in regulating cholesterol metabolism) to non-human primates. A single systemic administration of low clinically relevant amounts of SNALP-siRNA complexes caused a significant reduction in apo B mRNA and protein expression as well as a reduction in total cholesterol (Zimmermann et al., 2006). The nanoparticle-siRNA complexes were non-toxic and provided sustained knockdown of apo B for up to 11 days (Zimmermann et al., 2006). SNALPs have also been used to deliver siRNA to potently silence a gene involved in promoting aggressive tumor growth in an orthotopic mouse model of liver cancer (Judge et al., 2009). Collectively, these studies highlight the potential of using nanoparticle-siRNA complexes as novel therapeutics to treat human disease in the clinic.

Recently there has been intense research effort into the development of nanoparticles which contain targeting moieties (antibodies, peptides) conjugated to their surface to deliver siRNA specifically to tumor cells (Schroeder et al., 2010). Common examples of cell surface targets used for delivery to tumor cells include: transferrin receptor (TfR), folate receptor, and Arginine-Glycine-Aspartic ligands. It is to be noted that these cell surface receptors have been reported to be highly expressed in a number of different tumor types (Allen, 2002; Daniels et al., 2006; Basile et al., 2012). For instance, Pirollo et al. (2007) used nanoparticles (liposomes) which contained a TfR-antibody on their surface to complex siRNA against HER-2. Systemic delivery of the TfR-targeting nanoparticles resulted in more siRNA being delivered to pancreatic tumors in a murine xenograft model compared to nanoparticles without the TfR antibody (Pirollo et al., 2007). Moreover, delivery of the nanoparticle-siRNA complexes were able to silence HER-2 expression in the pancreatic tumor cells and increase sensitivity to gemcitabine (Pirollo et al., 2007). Studies have also taken interest in targeting nanoparticles carrying siRNA to the stroma of the pancreas. Of note, Ishiwatari et al. (2013) were able to target activated PSCs using vitamin A-coupled nanoparticles (liposomes) loaded with siRNA against collagen-specific chaperone protein gp46 (VA-lip-siRNAgp46) (Ishiwatari et al., 2013). VA-lip-siRNAgp46 complexes were actively taken up by PSCs and subsequent silencing of gp46 expression correlated to a significant decrease in PSC collagen secretion, which in turn resolved pancreatic fibrosis in a rat model of chronic pancreatitis (Ishiwatari et al., 2013). These promising findings point toward the therapeutic potential of nanoparticle-siRNA complexes in ablating the desmoplasia in pancreatic cancer.

Finally, siRNA-nanoparticle therapy based human clinical trials are underway to evaluate the safety and efficacy of systemic siRNA delivery using modified nanoparticles for the treatment of cancer (Burnett et al., 2011; Davidson and McCray, 2011). Most notably, a phase I clinical trial involving systemic siRNA administration to patients with solid tumors using a TfR-guided cyclodextrin-nanoparticle (CALAA-01) showed great promise (Davis et al., 2010). CALAA-01 is a nanoparticle-siRNA complex that was used to silence anti-ribonucleotide reductase (RRM2) in patients with metastatic melanoma. Results from this study showed that systemic administration of CALAA-01 reduced RRM2 mRNA and protein levels in tumor tissue collected via biopsy after adminstration (Davis et al., 2010). Another landmark phase I study recently reported the use of nanoparticles to simultaneously deliver two individual siRNAs targeting vascular endothelial growth factor (VEGF) and kinesin spindle protein (KSP) (Tabernero et al., 2013). The nanoparticle-siRNA complex later renamed ALN-VSP was administered bi-weekly (intravenously) and was found to be safe and well tolerated even up to 23 months (Tabernero et al., 2013). Importantly, results also showed disease control lasting more than 6 months and a complete regression of liver metastases in a patient with endometrial cancer (Tabernero et al., 2013).

## Concluding remarks

New strategies are needed to reduce the overall dismal prognosis and increase survival of pancreatic cancer patients. Nanomedicines offer great potential with benefits for diagnostics, imaging, and therapeutics. As our knowledge of pancreatic cancer becomes more complete, it is increasingly important for clinicians, biologists, and biochemical engineers to integrate novel ideas for the treatment of pancreatic cancer. Developing nanoparticle therapies based on the unique tumor microenvironment is crucial to the delivery of clinically relevant amounts of active therapeutics to the tumor site while by-passing various biological barriers. As highlighted in this review nanoparticles could be used to target tumor elements and stromal elements of pancreatic cancer. In summary, nanotechnology will have an important role in realizing the goal for early detection and personalized pancreatic cancer treatment for patients at different stages of disease, avoiding unnecessary toxicities associated with current treatment regimes and increasing patient survival.

### Conflict of interest statement

The authors declare that the research was conducted in the absence of any commercial or financial relationships that could be construed as a potential conflict of interest.

## Abbreviations

PanIN: Pancreatic intraductal neoplastic lesions
PSCs: pancreatic stellate cells
ECM: extracellular matrix
EUS: endoscopic ultrasonography
MRI: Magnetic Resonance Imaging
MRCP: Cholangiopancretography
EPR: enhanced permeability and retention
RNAi: RNA interference
RISC: RNA-induced silencing complex
siRNA: short-interfering RNA
Muc: Mucin
IONP: Iron oxide nanoparticle
TfR: Transferrin Receptor
SPION: Superparamagnetic Iron Oxide Nanoparticles.

## References

[B1] Aggarwal G. Ramachandran V. Javeed N. Arumugam T. Dutta S. Klee G. G. (2012). Adrenomedullin is up-regulated in patients with pancreatic cancer and causes insulin resistance in beta cells and mice. Gastroenterology 143, 1510–1517 e1511. 10.1053/j.gastro.2012.08.044 22960655 PMC3787599

[B2] Allen T. M. (2002). Ligand-targeted therapeutics in anticancer therapy. Nat. Rev. Cancer 2, 750–763 10.1038/nrc903 12360278

[B3] Apte M. V. Park S. Phillips P. A. Santucci N. Goldstein D. Kumar R. K. (2004). Desmoplastic reaction in pancreatic cancer: role of pancreatic stellate cells. Pancreas 29, 179–187 10.1097/00006676-200410000-00002 15367883

[B4] Bachem M. G. Schunemann M. Ramadani M. Siech M. Beger H. Buck A. (2005). Pancreatic carcinoma cells induce fibrosis by stimulating proliferation and matrix synthesis of stellate cells. Gastroenterology 128, 907–921 10.1053/j.gastro.2004.12.036 15825074

[B5] Baigude H. McCarroll J. Yang C. S. Swain P. M. Rana T. M. (2007). Design and creation of new nanomaterials for therapeutic RNAi. ACS Chem. Biol. 2, 237–241 10.1021/cb7000582 17432823

[B6] Baigude H. Rana T. M. (2009). Delivery of therapeutic RNAi by nanovehicles. Chembiochem 10, 2449–2454 10.1002/cbic.200900252 19688785 PMC3517099

[B7] Barenholz Y. (2012). Doxil(R)-the first FDA-approved nano-drug: lessons learned. J. Control. Release 160, 117–134 10.1016/j.jconrel.2012.03.020 22484195

[B8] Basile L. Pignatello R. Passirani C. (2012). Active targeting strategies for anticancer drug nanocarriers. Curr. Drug Deliv. 9, 255–268 10.2174/156720112800389089 22452402

[B9] Blanco E. Hsiao A. Mann A. P. Landry M. G. Meric-Bernstam F. Ferrari M. (2011). Nanomedicine in cancer therapy: innovative trends and prospects. Cancer Sci. 102, 1247–1252 10.1111/j.1349-7006.2011.01941.x 21447010 PMC11158341

[B10] Boyer C. Bulmus V. Liu J. Davis T. P. (2010). The design and utility of polymer-stabilized iron-oxide nanoparticles for nanomedicine aaplications. NPG Asia Mater. 2, 22–30 10.1038/asiamat.2010.6

[B11] Brand R. (2001). The diagnosis of pancreatic cancer. Cancer J. 7, 287–297 11561605

[B12] Burnett J. C. Rossi J. J. Tiemann K. (2011). Current progress of siRNA/shRNA therapeutics in clinical trials. Biotechnol. J. 6, 1130–1146 10.1002/biot.201100054 21744502 PMC3388104

[B13] Cabral H. Murakami M. Hojo H. Terada Y. Kano M. R. Chung U. I. (2013). Targeted therapy of spontaneous murine pancreatic tumors by polymeric micelles prolongs survival and prevents peritoneal metastasis. Proc. Natl. Acad. Sci. U.S.A. 110, 11397–11402 10.1073/pnas.1301348110 23801758 PMC3710858

[B14] Canto M. I. Hruban R. H. Fishman E. K. Kamel I. R. Schulick R. Zhang Z. (2012). Frequent detection of pancreatic lesions in asymptomatic high-risk individuals. Gastroenterology 142, 796–804 quiz: e714–e795. 10.1053/j.gastro.2012.01.005 22245846 PMC3321068

[B15] Cao Y. C. (2008). Nanomaterials for biomedical applications. Nanomedicine 3, 467–469 10.2217/17435889.3.4.467 18694308

[B16] Chang H. K. Ishikawa F. N. Zhang R. Datar R. Cote R. J. Thompson M. E. (2011). Rapid, label-free, electrical whole blood bioassay based on nanobiosensor systems. ACS Nano 5, 9883–9891 10.1021/nn2035796 22066492

[B17] Cheng W. Ping Y. Zhang Y. Chuang K. H. Liu Y. (2013). Magnetic resonance imaging (MRI) contrast agents for tumor diagnosis. J. Healthc. Eng. 4, 23–45 10.1260/2040-2295.4.1.23 23502248

[B18] Chikkaveeraiah B. V. Bhirde A. A. Morgan N. Y. Eden H. S. Chen X. (2012). Electrochemical immunosensors for detection of cancer protein biomarkers. ACS Nano 6, 6546–6561 10.1021/nn3023969 22835068 PMC3429657

[B19] Daniels T. R. Delgado T. Helguera G. Penichet M. L. (2006). The transferrin receptor part II: targeted delivery of therapeutic agents into cancer cells. Clin. Immunol. 121, 159–176 10.1016/j.clim.2006.06.006 16920030

[B20] Davidson B. L. McCray P. B. Jr. (2011). Current prospects for RNA interference-based therapies. Nat. Rev. Genet. 12, 329–340 10.1038/nrg2968 21499294 PMC7097665

[B21] Davis M. E. Zuckerman J. E. Choi C. H. Seligson D. Tolcher A. Alabi C. A. (2010). Evidence of RNAi in humans from systemically administered siRNA via targeted nanoparticles. Nature 464, 1067–1070 10.1038/nature08956 20305636 PMC2855406

[B22] Eck W. Craig G. Sigdel A. Ritter G. Old L. J. Tang L. (2008). PEGylated gold nanoparticles conjugated to monoclonal F19 antibodies as targeted labeling agents for human pancreatic carcinoma tissue. ACS Nano 2, 2263–2272 10.1021/nn800429d 19206392

[B23] Erkan M. Hausmann S. Michalski C. W. Fingerle A. A. Dobritz M. Kleeff J. (2012a). The role of stroma in pancreatic cancer: diagnostic and therapeutic implications. Nat. Rev. Gastroenterol. Hepatol. 9, 454–467 10.1038/nrgastro.2012.115 22710569

[B24] Erkan M. Hausmann S. Michalski C. W. Schlitter A. M. Fingerle A. A. Dobritz M. (2012b). How fibrosis influences imaging and surgical decisions in pancreatic cancer. Front. Physiol. 3:389 10.3389/fphys.2012.00389 23060813 PMC3462403

[B25] Erkan M. Kleeff J. Gorbachevski A. Reiser C. Mitkus T. Esposito I. (2007). Periostin creates a tumor-supportive microenvironment in the pancreas by sustaining fibrogenic stellate cell activity. Gastroenterology 132, 1447–1464 10.1053/j.gastro.2007.01.031 17408641

[B26] Ferrari M. (2005). Cancer nanotechnology: opportunities and challenges. Nat. Rev. Cancer 5, 161–171 10.1038/nrc1566 15738981

[B27] Godin B. Tasciotti E. Liu X. Serda R. E. Ferrari M. (2011). Multistage nanovectors: from concept to novel imaging contrast agents and therapeutics. Acc. Chem. Res. 44, 979–989 10.1021/ar200077p 21902173 PMC3204797

[B28] Goggins M. (2005). Molecular markers of early pancreatic cancer. J. Clin. Oncol. 23, 4524–4531 10.1200/JCO.2005.19.711 16002843

[B29] Hahn M. A. Singh A. K. Sharma P. Brown S. C. Moudgil B. M. (2011). Nanoparticles as contrast agents for in-vivo bioimaging: current status and future perspectives. Anal. Bioanal. Chem. 399, 3–27 10.1007/s00216-010-4207-5 20924568

[B30] Hart P. A. Kamada P. Rabe K. G. Srinivasan S. Basu A. Aggarwal G. (2011). Weight loss precedes cancer-specific symptoms in pancreatic cancer-associated diabetes mellitus. Pancreas 40, 768–772 10.1097/MPA.0b013e318220816a 21654538 PMC3118443

[B31] Harvey S. R. Hurd T. C. Markus G. Martinick M. Penetrante R. M. Tan D. (2003). Evaluation of urinary plasminogen activator, its receptor, matrix metalloproteinase-9, and von Willebrand factor in pancreatic cancer. Clin. Cancer Res. 9, 4935–4943 14581368

[B32] Heinemann V. Reni M. Ychou M. Richel D. J. Macarulla T. Ducreux M. (2013). Tumour-stroma interactions in pancreatic ductal adenocarcinoma: rationale and current evidence for new therapeutic strategies. Cancer Treat. Rev. 40, 118–128 10.1016/j.ctrv.2013.04.004 23849556

[B33] Hekimoglu K. Ustundag Y. Dusak A. Erdem Z. Karademir B. Aydemir S. (2008). MRCP vs. ERCP in the evaluation of biliary pathologies: review of current literature. J. Dig. Dis. 9, 162–169 10.1111/j.1751-2980.2008.00339.x 18956595

[B34] Hidalgo M. (2010). Pancreatic cancer. N. Engl. J. Med. 362, 1605–1617 10.1056/NEJMra0901557 20427809

[B35] Holzapfel K. Reiser-Erkan C. Fingerle A. A. Erkan M. Eiber M. J. Rummeny E. J. (2011). Comparison of diffusion-weighted MR imaging and multidetector-row CT in the detection of liver metastases in patients operated for pancreatic cancer. Abdom. Imaging 36, 179–184 10.1007/s00261-010-9633-5 20563868

[B36] Hu M. Yan J. He Y. Lu H. Weng L. Song S. (2010). Ultrasensitive, multiplexed detection of cancer biomarkers directly in serum by using a quantum dot-based microfluidic protein chip. ACS Nano 4, 488–494 10.1021/nn901404h 20041634

[B37] Huang H. C. Barua S. Sharma G. Dey S. K. Rege K. (2011). Inorganic nanoparticles for cancer imaging and therapy. J. Control. Release 155, 344–357 10.1016/j.jconrel.2011.06.004 21723891

[B38] Hyodo T. Kumano S. Kushihata F. Okada M. Hirata M. Tsuda T. (2012). CT and MR cholangiography: advantages and pitfalls in perioperative evaluation of biliary tree. Br. J. Radiol. 85, 887–896 10.1259/bjr/21209407 22422383 PMC3474084

[B39] Ishiwatari H. Sato Y. Murase K. Yoneda A. Fujita R. Nishita H. (2013). Treatment of pancreatic fibrosis with siRNA against a collagen-specific chaperone in vitamin A-coupled liposomes. Gut 62, 1328–1339 10.1136/gutjnl-2011-301746 23172890

[B40] Jain R. K. Stylianopoulos T. (2010). Delivering nanomedicine to solid tumors. Nat. Rev. Clin. Oncol. 7, 653–664 10.1038/nrclinonc.2010.139 20838415 PMC3065247

[B41] Jemal A. Bray F. Center M. M. Ferlay J. Ward E. Forman D. (2011). Global cancer statistics. CA Cancer J. Clin. 61, 69–90 10.3322/caac.20107 21296855

[B42] Judge A. D. Robbins M. Tavakoli I. Levi J. Hu L. Fronda A. (2009). Confirming the RNAi-mediated mechanism of action of siRNA-based cancer therapeutics in mice. J. Clin. Invest. 119, 661–673 10.1172/JCI37515 19229107 PMC2648695

[B43] Kaida S. Cabral H. Kumagai M. Kishimura A. Terada Y. Sekino M. (2010). Visible drug delivery by supramolecular nanocarriers directing to single-platformed diagnosis and therapy of pancreatic tumor model. Cancer Res. 70, 7031–7041 10.1158/0008-5472.CAN-10-0303 20685894

[B44] Kaur S. Kumar S. Momi N. Sasson A. R. Batra S. K. (2013). Mucins in pancreatic cancer and its microenvironment. Nat. Rev. Gastroenterol. Hepatol. 10, 607–620 10.1038/nrgastro.2013.120 23856888 PMC3934431

[B45] Kelly K. A. Bardeesy N. Anbazhagan R. Gurumurthy S. Berger J. Alencar H. (2008). Targeted nanoparticles for imaging incipient pancreatic ductal adenocarcinoma. PLoS Med. 5:e85 10.1371/journal.pmed.0050085 18416599 PMC2292750

[B46] Kim B. H. Lee N. Kim H. An K. Park Y. I. Choi Y. (2011). Large-scale synthesis of uniform and extremely small-sized iron oxide nanoparticles for high-resolution T1 magnetic resonance imaging contrast agents. J. Am. Chem. Soc. 133, 12624–12631 10.1021/ja203340u 21744804

[B47] Kirui D. K. Khalidov I. Wang Y. Batt C. A. (2013). Targeted near-IR hybrid magnetic nanoparticles for in vivo cancer therapy and imaging. Nanomedicine 9, 702–711 10.1016/j.nano.2012.11.009 23219875

[B48] Komar G. Kauhanen S. Liukko K. Seppanen M. Kajander S. Ovaska J. (2009). Decreased blood flow with increased metabolic activity: a novel sign of pancreatic tumor aggressiveness. Clin. Cancer Res. 15, 5511–5517 10.1158/1078-0432.CCR-09-0414 19706808

[B49] Koong A. C. Mehta V. K. Le Q. T. Fisher G. A. Terris D. J. Brown J. M. (2000). Pancreatic tumors show high levels of hypoxia. Int. J. Radiat. Oncol. Biol. Phys. 48, 919–922 10.1016/S0360-3016(00)00803-8 11072146

[B50] Kumagai M. Sarma T. K. Cabral H. Kaida S. Sekino M. Herlambang N. (2010). Enhanced in vivo magnetic resonance imaging of tumors by PEGylated iron-oxide-gold core-shell nanoparticles with prolonged blood circulation properties. Macromol. Rapid Commun. 31, 1521–1528 10.1002/marc.201000341 21567561

[B51] Leamon C. P. Lovejoy C. D. Nguyen B. (2013). Patient selection and targeted treatment in the management of platinum-resistant ovarian cancer. Pharmgenomics Pers. Med. 6, 113–125 10.2147/PGPM.S24943 24109193 PMC3792616

[B52] Lee G. Y. Qian W. P. Wang L. Wang Y. A. Staley C. A. Satpathy M. (2013). Theranostic nanoparticles with controlled release of gemcitabine for targeted therapy and MRI of pancreatic cancer. ACS Nano 7, 2078–2089 10.1021/nn3043463 23402593 PMC3609912

[B53] Li J. Wientjes M. G. Au J. L. (2010). Pancreatic cancer: pathobiology, treatment options, and drug delivery. AAPS J. 12, 223–232 10.1208/s12248-010-9181-5 20198462 PMC2844509

[B54] Ma W. W. Hidalgo M. (2013). The winning formulation: the development of Paclitaxel in pancreatic cancer. Clin. Cancer Res. 19, 5572–5579 10.1158/1078-0432.CCR-13-1356 23918602

[B55] Maeda H. Wu J. Sawa T. Matsumura Y. Hori K. (2000). Tumor vascular permeability and the EPR effect in macromolecular therapeutics: a review. J. Control. Release 65, 271–284 10.1016/S0168-3659(99)00248-5 10699287

[B56] Malhotra R. Patel V. Chikkaveeraiah B. V. Munge B. S. Cheong S. C. Zain R. B. (2012). Ultrasensitive detection of cancer biomarkers in the clinic by use of a nanostructured microfluidic array. Anal. Chem. 84, 6249–6255 10.1021/ac301392g 22697359 PMC3418660

[B57] Matsumura Y. Maeda H. (1986). A new concept for macromolecular therapeutics in cancer chemotherapy: mechanism of tumoritropic accumulation of proteins and the antitumor agent smancs. Cancer Res. 46, 6387–6392 2946403

[B58] Melancon M. P. Stafford R. J. Li C. (2012). Challenges to effective cancer nanotheranostics. J. Control. Release 164, 177–182 10.1016/j.jconrel.2012.07.045 22906841 PMC3504179

[B59] Misek D. E. Patwa T. H. Lubman D. M. Simeone D. M. (2007). Early detection and biomarkers in pancreatic cancer. J. Natl. Compr. Canc. Netw. 5, 1034–1041 18053427 10.6004/jnccn.2007.0086

[B60] Nagaraj V. J. Aithal S. Eaton S. Bothara M. Wiktor P. Prasad S. (2010). NanoMonitor: a miniature electronic biosensor for glycan biomarker detection. Nanomedicine (Lond). 5, 369–378 10.2217/nnm.10.11 20394531

[B61] Namiki Y. Fuchigami T. Tada N. Kawamura R. Matsunuma S. Kitamoto Y. (2011). Nanomedicine for cancer: lipid-based nanostructures for drug delivery and monitoring. Acc. Chem. Res. 44, 1080–1093 10.1021/ar200011r 21786832

[B62] Neesse A. Michl P. Frese K. K. Feig C. Cook N. Jacobetz M. A. (2011). Stromal biology and therapy in pancreatic cancer. Gut 60, 861–868 10.1136/gut.2010.226092 20966025

[B63] Olive K. P. Jacobetz M. A. Davidson C. J. Gopinathan A. McIntyre D. Honess D. (2009). Inhibition of Hedgehog signaling enhances delivery of chemotherapy in a mouse model of pancreatic cancer. Science 324, 1457–1461 10.1126/science.1171362 19460966 PMC2998180

[B64] Pearce T. R. Shroff K. Kokkoli E. (2012). Peptide targeted lipid nanoparticles for anticancer drug delivery. Adv. Mater. 24, 3803–3822, 3710. 10.1002/adma.201200832 22674563

[B65] Phillips P. (2012). Pancretic cancer and tumor microenvironment, in Pancreatic Stellate Cells and Fibrosis, Chapter 3, eds Grippo P. J. Munshi H. G. (Trivandrum: Transworld Research Network). Available online at: http://www.ncbi.nlm.nih.gov/books/NBK98937/22876388

[B66] Pirollo K. F. Rait A. Zhou Q. Hwang S. H. Dagata J. A. Zon G. (2007). Materializing the potential of small interfering RNA via a tumor-targeting nanodelivery system. Cancer Res. 67, 2938–2943 10.1158/0008-5472.CAN-06-4535 17409398

[B67] Prabhu P. Patravale V. (2012). The upcoming field of theranostic nanomedicine: an overview. J. Biomed. Nanotechnol. 8, 859–882 10.1166/jbn.2012.1459 23029995

[B68] Provenzano P. P. Cuevas C. Chang A. E. Goel V. K. Von Hoff D. D. Hingorani S. R. (2012). Enzymatic targeting of the stroma ablates physical barriers to treatment of pancreatic ductal adenocarcinoma. Cancer Cell 21, 418–429 10.1016/j.ccr.2012.01.007 22439937 PMC3371414

[B69] Rachagani S. Torres M. P. Kumar S. Haridas D. Baine M. Macha M. A. (2012). Mucin (Muc) expression during pancreatic cancer progression in spontaneous mouse model: potential implications for diagnosis and therapy. J. Hematol. Oncol. 5, 68 10.1186/1756-8722-5-68 23102107 PMC3511181

[B70] Rana T. M. (2007). Illuminating the silence: understanding the structure and function of small RNAs. Nat. Rev. Mol. Cell Biol. 8, 23–36 10.1038/nrm2085 17183358

[B71] Remmers N. Anderson J. M. Linde E. M. Dimaio D. J. Lazenby A. J. Wandall H. H. (2013). Aberrant expression of mucin core proteins and o-linked glycans associated with progression of pancreatic cancer. Clin. Cancer Res. 19, 1981–1993 10.1158/1078-0432.CCR-12-2662 23446997 PMC3873635

[B72] Roberts T. P. (1997). Physiologic measurements by contrast-enhanced MR imaging: expectations and limitations. J. Magn. Reson. Imaging 7, 82–90 10.1002/jmri.1880070112 9039597

[B73] Sah R. P. Nagpal S. J. Mukhopadhyay D. Chari S. T. (2013). New insights into pancreatic cancer-induced paraneoplastic diabetes. Nat. Rev. Gastroenterol. Hepatol. 10, 423–433 10.1038/nrgastro.2013.49 23528347 PMC3932322

[B74] Schroeder A. Heller D. A. Winslow M. M. Dahlman J. E. Pratt G. W. Langer R. (2011). Treating metastatic cancer with nanotechnology. Nat. Rev. Cancer 12, 39–50 10.1038/nrc3180 22193407

[B75] Schroeder A. Levins C. G. Cortez C. Langer R. Anderson D. G. (2010). Lipid-based nanotherapeutics for siRNA delivery. J. Intern. Med. 267, 9–21 10.1111/j.1365-2796.2009.02189.x 20059641 PMC5308083

[B76] Siegel R. Naishadham D. Jemal A. (2013). Cancer statistics, 2013. CA Cancer J. Clin. 63, 11–30 10.3322/caac.21166 23335087

[B77] Singh S. Sharma A. Robertson G. P. (2012). Realizing the clinical potential of cancer nanotechnology by minimizing toxicologic and targeted delivery concerns. Cancer Res. 72, 5663–5668 10.1158/0008-5472.CAN-12-1527 23139207 PMC3616627

[B78] Slavin J. Ghaneh P. Jones L. Sutton R. Hartley M. Neoptolemos J. P. (1999). The future of surgery for pancreatic cancer. Ann. Oncol. 10(Suppl. 4), 285–290 10.1093/annonc/10.suppl_4.S285 10436842

[B79] Su J. Baigude H. McCarroll J. Rana T. M. (2011). Silencing microRNA by interfering nanoparticles in mice. Nucleic Acids Res. 39, e38 10.1093/nar/gkq1307 21212128 PMC3064800

[B80] Tabernero J. Shapiro G. I. Lorusso P. M. Cervantes A. Schwartz G. K. Weiss G. J. (2013). First-in-humans trial of an RNA interference therapeutic targeting VEGF and KSP in cancer patients with liver involvement. Cancer Discov. 3, 406–417 10.1158/2159-8290.CD-12-0429 23358650

[B81] Vincent A. Herman J. Schulick R. Hruban R. H. Goggins M. (2011). Pancreatic cancer. Lancet 378, 607–620 10.1016/S0140-6736(10)62307-0 21620466 PMC3062508

[B82] Von Hoff D. D. Ervin T. Arena F. P. Chiorean E. G. Infante J. Moore M. (2013). Increased survival in pancreatic cancer with nab-paclitaxel plus gemcitabine. N. Engl. J. Med. 369, 1691–1703 10.1056/NEJMoa1304369 24131140 PMC4631139

[B83] Von Hoff D. D. Ramanathan R. K. Borad M. J. Laheru D. A. Smith L. S. Wood T. E. (2011). Gemcitabine plus nab-paclitaxel is an active regimen in patients with advanced pancreatic cancer: a phase I/II trial. J. Clin. Oncol. 29, 4548–4554 10.1200/JCO.2011.36.5742 21969517 PMC3565012

[B84] Vonlaufen A. Phillips P. A. Xu Z. Goldstein D. Pirola R. C. Wilson J. S. (2008). Pancreatic stellate cells and pancreatic cancer cells: an unholy alliance. Cancer Res. 68, 7707–7710 10.1158/0008-5472.CAN-08-1132 18829522

[B85] Wonganan P. Lansakara P. D. Zhu S. Holzer M. Sandoval M. A. Warthaka M. (2013). Just getting into cells is not enough: mechanisms underlying 4-(N)-stearoyl gemcitabine solid lipid nanoparticle's ability to overcome gemcitabine resistance caused by RRM1 overexpression. J. Control. Release 169, 17–27 10.1016/j.jconrel.2013.03.033 23570983 PMC3683387

[B86] Xu Z. Vonlaufen A. Phillips P. A. Fiala-Beer E. Zhang X. Yang L. (2010). Role of pancreatic stellate cells in pancreatic cancer metastasis. Am. J. Pathol. 177, 2585–2596 10.2353/ajpath.2010.090899 20934972 PMC2966814

[B87] Yang L. Mao H. Cao Z. Wang Y. A. Peng X. Wang X. (2009). Molecular imaging of pancreatic cancer in an animal model using targeted multifunctional nanoparticles. Gastroenterology 136, 1514–1525 e1512. 10.1053/j.gastro.2009.01.006 19208341 PMC3651919

[B88] Zhu S. Wonganan P. Lansakara P. D. O'mary H. L. Li Y. Cui Z. (2013). The effect of the acid-sensitivity of 4-(N)-stearoyl gemcitabine-loaded micelles on drug resistance caused by RRM1 overexpression. Biomaterials 34, 2327–2339 10.1016/j.biomaterials.2012.11.053 23261218 PMC3552003

[B89] Zimmermann T. S. Lee A. C. Akinc A. Bramlage B. Bumcrot D. Fedoruk M. N. (2006). RNAi-mediated gene silencing in non-human primates. Nature 441, 111–114 10.1038/nature04688 16565705

